# Hidradenitis Suppurativa on TikTok: Representation, Treatment Messaging, and Psychosocial Themes

**DOI:** 10.7759/cureus.113677

**Published:** 2026-07-30

**Authors:** Christine Lee, Guang Orestes, Victoria Farley, Bertille Mavegam Tango

**Affiliations:** 1 Dermatology, Kirk Kerkorian School of Medicine at University of Nevada, Las Vegas, USA; 2 Dermatology, Vivida Dermatology, Las Vegas, USA; 3 Academic Affairs and Education, Kirk Kerkorian School of Medicine at University of Nevada, Las Vegas, USA

**Keywords:** hidradenitis suppurativa, patient education, psychosocial, skin of color, tiktok

## Abstract

Background: Hidradenitis suppurativa (HS) disproportionately affects women and individuals with skin of color (SOC) and is associated with a significant psychosocial burden. TikTok has become a major platform for HS storytelling, yet representation, treatment messaging, and psychosocial factors remain poorly characterized.

Objective: The objective of this study is to characterize creator demographics, SOC representation, treatment discussions, and psychosocial themes in highly visible HS TikTok videos.

Methods: We performed a cross-sectional content analysis of the top 200 TikTok videos under four HS-related hashtags. After removing duplicates and unrelated posts, 133 videos were coded for creator type, content format, gender, Fitzpatrick type, treatment, and psychosocial themes. A 20% subsample was dual-coded to assess interrater reliability. Engagement rates were calculated, and group differences were assessed using t-tests and one-way analysis of variance.

Results: Most videos were created by patients (90%), with women representing the majority of creators (90%). Most videos were testimonials (80%). Representation of Fitzpatrick IV-VI was high (57%). Treatments discussed included guideline-supported therapies (24%), over-the-counter adjuncts (29%), and alternative remedies (19%). Psychosocial themes of empowerment (88%) and mental health (36%) were common. There was no significant difference in engagement across creator type, skin tone, or content format.

Conclusion: HS TikTok videos are dominated by women and SOC creators sharing testimonials about lived experiences. While psychosocial support is prominent, guideline-based treatment information and dermatology provider input are limited, highlighting an opportunity for clinicians to share accurate and inclusive HS education on social media.

## Introduction

Hidradenitis suppurativa (HS) is a multifactorial, chronic inflammatory skin disease characterized by painful nodules, abscesses, draining sinus tracts, and scarring, most commonly affecting areas rich in apocrine glands, including the axillae, groin, perineal region, and inframammary folds [1-3]. HS has an estimated global prevalence of 0.00033-4.1% [2]. Frequent recurrence, chronic pain, malodorous drainage, and visible scarring contribute to impaired quality of life and social stigma [2]. Beyond its cutaneous manifestations, HS is associated with substantial psychosocial burden, including high rates of depression, social isolation, and suicidal ideation [2,4]. HS disproportionately affects individuals with skin of color (SOC), particularly Black women, who experience higher disease prevalence and greater disease severity at presentation [5,6]. In a 48-million-patient analysis in the United States, African American patients had a prevalence of 296 per 100,000 compared with 95 per 100,000 among White patients [7]. Barriers such as delayed diagnosis, underrecognition of disease severity in darker skin tones, and limited representation in dermatology literature contribute to ongoing disparities in care. These inequities may result in delayed treatment initiation and prolonged disease burden.

In the setting of unmet clinical needs and delayed care, many patients turn to social media for health information, community support, and validation. Due to its short-form, visually driven content and algorithmic promotion of highly engaging narratives, TikTok has emerged as a major platform for disease storytelling and peer support [8]. Existing literature suggests that HS content on TikTok is largely generated by patients, with limited participation from healthcare professionals (HCPs) and generally low educational quality and accuracy [9]. For example, Rehman and colleagues examined the quality of popular HS content on TikTok by analyzing the top 100 English-language videos using the DISCERN instrument. They reported that the overall quality of information was poor, with most videos created by non-physicians, which scored significantly lower than physician-created content [8]. In another study, Wolinska and colleagues compared HS content across Instagram, TikTok, and Facebook by analyzing the top 50 posts per platform tagged with #HidradenitisSuppurativa. They reported that TikTok accounts had substantially higher follower counts than Instagram and Facebook and that HS-related content was predominantly generated by patients rather than medical professionals [9]. Saini and colleagues performed a qualitative thematic analysis of #HidradenitisSuppurativa, highlighting the platform’s role in shaping patient perceptions and support networks [10]. Zadu and colleagues conducted a cross-sectional analysis of the top 50 videos for “hidradenitis suppurativa in Black skin” and found that videos were predominantly patient testimonials [11]. While these studies have provided important insights into psychosocial experiences and SOC representation, they are limited in their focus on individual content domains or single methodological approaches. Questions remain regarding how HS is portrayed when considering creator demographics, broader SOC representation, treatment discussions, and psychosocial themes collectively within a unified analytical framework.

Therefore, we conducted a cross-sectional analysis of top-ranked HS-related TikTok videos under selected hashtags. The primary aim was to characterize creator demographics, SOC representation across Fitzpatrick skin types, guideline-supported treatment discussions, and mental health and empowerment themes within a single quantitative framework.

## Materials and methods

Study design

We conducted a cross-sectional content analysis of publicly available TikTok videos related to HS. This study received an Institutional Review Board exemption because no human subjects were directly involved and only publicly available metadata were collected.

Video selection

A new TikTok account was created prior to data collection to minimize algorithmic bias. Videos were searched on September 18, 2025, using four prespecified hashtags commonly associated with HS: #HidradenitisSuppurativa, #HSwarrior, #HSjourney, and #HScommunity. Searches were conducted using TikTok’s default ranking algorithm, which prioritizes engagement and relevance. Results were not filtered or sorted manually.

We analyzed the top 50 videos per hashtag (n = 200) to reflect real-world user exposure, as TikTok users predominantly engage with high-ranking content and rarely scroll extensively through hashtag search results. This approach captures the most visible and influential videos prioritized by the platform’s algorithm and is consistent with other social media-based cross-sectional studies [9,12,13]. URLs were compiled into a master dataset. Two independent reviewers then screened each video for eligibility. Duplicates were removed using Google Sheets’ duplicate detection tool (n = 31). 

Inclusion criteria were HS-related educational, testimonial, or treatment-focused content; English language; and public accessibility at the time of review. Exclusion criteria were irrelevant content, videos lacking both audio and caption content, and duplicates.

After screening, 133 videos met inclusion criteria. Three videos later became private or were deleted. These videos were retained for qualitative coding but excluded from engagement metrics analysis.

Coding framework and variable definitions

A standardized coding sheet with predefined operational definitions was used for all included videos. Before independent coding, reviewers reviewed the coding framework and category definitions to promote consistent application of coding criteria. Ambiguous cases and coding disagreements were resolved through discussion until consensus was reached.

Creator Type

Creators were categorized based on publicly available information from the video, including the creator’s TikTok username, profile biography, and verbal self-identification when present. Creator categories included the following:

Patient: An individual who self-identified as having HS without formal medical training.

Dermatology healthcare professional (dermatology HCP): A licensed medical professional who explicitly identified as a dermatologist (MD/DO), dermatology mid-level provider (physician assistant or nurse practitioner), or dermatology trainee (resident or fellow).

Other healthcare professional (other HCP): A licensed HCP who did not specialize in dermatology but produced HS-related content, including plastic surgeons, nurses, and other health professionals.

Advocacy: A group or individual acting in an organized advocacy capacity, including nonprofit organizations, patient advocacy groups, awareness campaigns, or HS support initiatives.

Other: Creators who met inclusion criteria but could not be confidently classified into the above categories based on available information.

Content Format

Each video was categorized by its primary purpose. Content categories included the following:

Testimonial: Content in which the creator shared personal experiences with HS, including disease onset, symptom burden, treatment journeys, flare coping strategies, or psychosocial impact. These videos were typically first-person narratives centered on lived experiences rather than formal medical instruction or product endorsement.

Educational: Videos primarily intended to convey medical or health information about HS, including explanations of disease pathophysiology, risk factors, diagnosis, and treatment options. Educational content could be created by patients or HCPs and did not require professional credentials, provided the primary purpose was informational.

Promotional: Content primarily intended to market or endorse a product, service, or brand related to HS management. This included paid or unpaid advertisements, affiliate marketing, sponsored content, product reviews, or explicit calls to purchase or use specific treatments, such as topical agents, supplements, or skincare products. Videos were classified as promotional regardless of creator type when there was evident commercial intent.

Advocacy: Content focused on raising awareness, reducing stigma, encouraging community engagement, or supporting initiatives related to HS.

Other: Videos that met inclusion criteria but did not clearly align with the above categories.

Demographics

Perceived gender and race/ethnicity were coded based on visual cues or self-identification when available. These variables were classified as perceived rather than self-reported, acknowledging the inherent limitations and potential for misclassification when assessing demographic characteristics from publicly available social media content.

Fitzpatrick Skin Type

Fitzpatrick skin type is a commonly used dermatologic classification system that categorizes skin based on response to sun exposure, specifically the tendency to burn and the ability to tan. The scale ranges from type I to type VI, with type I representing very lightly pigmented skin that always burns and never tans and type VI representing deeply pigmented skin with minimal to no burning [14]. Fitzpatrick classification is widely used in research and clinical practice, including assessment of skin cancer risk, determination of therapeutic ultraviolet dosing, and prediction of outcomes for dermatologic procedures [14].

In this study, perceived Fitzpatrick skin type was assigned through visual assessment of publicly available TikTok videos and categorized as Fitzpatrick I-III, mixed, and IV-VI for analysis. Mixed was defined as intermediate between Fitzpatrick III and IV. This approach aligns with prior social media-based skin color representation studies in which direct participant contact, self-report, and objective pigmentation measurements were not feasible [15,16]. Videos with multiple individuals of different Fitzpatrick types, or those with insufficient visual information to allow classification, were categorized as undetermined.

Treatment Categories

Dermatology-supported treatments were selected based on the 2019 North American clinical management guidelines for hidradenitis suppurativa [17]. Categories included the following:

Topicals: Clindamycin, zinc pyrithione, chlorhexidine, resorcinol, triamcinolone, benzoyl peroxide, and dapsone.

Systemic antibiotics: Tetracyclines, rifampin plus clindamycin, triple therapy, dapsone, and ertapenem.

Hormonal therapies: Antiandrogen contraceptives, spironolactone, metformin, and finasteride.

Retinoids: Isotretinoin, acitretin, and alitretinoin.

Systemic immunomodulators: Cyclosporine, systemic steroids, and colchicine in combination with minocycline.

Biologics: Adalimumab, infliximab, anakinra, ustekinumab, etanercept, and golimumab.

Procedures/surgery: Deroofing, local or wide excision, laser treatment, and incision and drainage.

Over-the-counter (OTC) adjunctive products were defined as wound dressings, absorbent pads, non-guideline antiseptic washes, and OTC pain medications. Alternative or non-evidence-based remedies were defined as commonly used remedies such as witch hazel, herbal teas, essential oils, antiseptic solutions, and non-medication salves.

Psychosocial Themes

Mental health themes included discussion of depression, anxiety, stigma, and mental health therapy.

Survivor/empowerment themes included discussion of resiliency, coping, self-acceptance, body positivity, and encouragement directed toward oneself or others in the context of living with HS.

Lifestyle measures included discussion of weight management, smoking cessation, stress reduction strategies, hygiene routines, wound care, and exercise.

Mentions of HS triggers included diet, hormones, stress, menstrual cycle, friction, and environmental factors.

Reliability testing

To assess coding reliability, 20% of the included videos were randomly selected and independently dual-coded by two reviewers. Inter-rater agreement was calculated using Cohen’s kappa, which quantifies the degree of agreement beyond chance. The statistic ranges from 0, indicating agreement no better than chance, to 1, indicating perfect agreement [18]. Kappa values for each coded domain are reported in the Results section.

Statistical analysis

Descriptive statistics were used to summarize video characteristics, creator demographics, content types, and psychosocial themes. Engagement rate was calculated using the following formula:

Engagement rate = (likes + comments + saves + shares) / views

Videos with missing engagement metrics were excluded from quantitative analyses but retained for qualitative coding. Independent Welch’s t-tests were used to compare engagement by creator type (HCP vs. non-HCP) and Fitzpatrick skin type (Fitzpatrick I-III vs. IV-VI). One-way analysis of variance (ANOVA) was used to assess differences across video content formats including education, testimonial, and product recommendation. Content formats with small subgroup sizes were excluded from ANOVA. Statistical analysis was performed using Google Sheets. Statistical significance was set at p < 0.05.

## Results

Video selection

**Figure 1:**
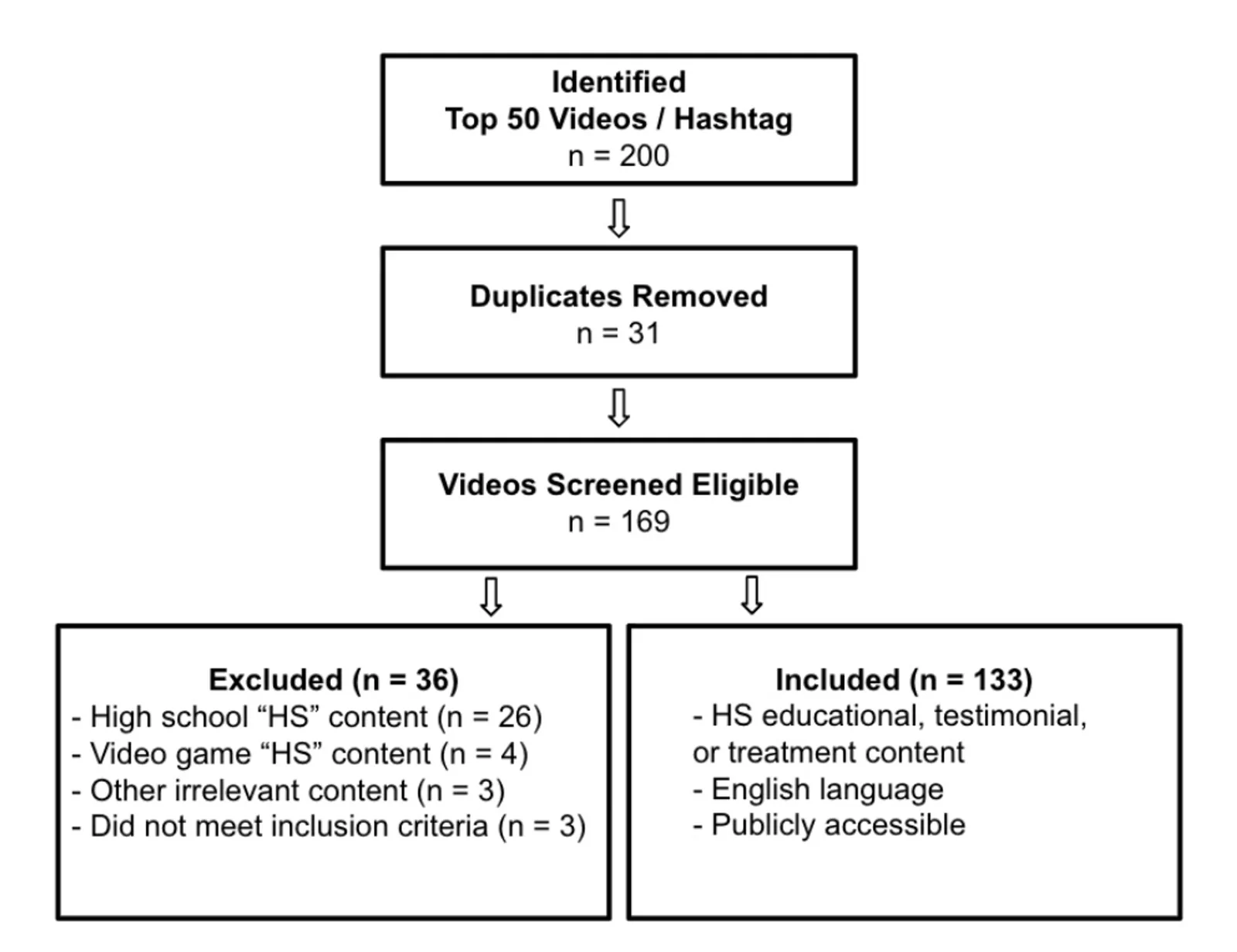
Flow diagram of the TikTok video selection process. Data are presented as n. HS: Hidradenitis suppurativa

Of 200 videos initially identified, 31 duplicates were removed, leaving 169 unique videos for screening. Thirty-six videos were excluded for irrelevance. A total of 133 videos (79%) were included in the final analysis (Figure 1).

Three videos subsequently became private or were deleted; these were retained for qualitative content coding but excluded from engagement metric analysis.

Interrater reliability

Interrater reliability ranged from substantial to perfect across coding domains. Agreement was high for inclusion criteria (κ = 0.90). Strong agreement was observed for creator type (κ = 0.80) and Fitzpatrick skin type grouping (κ = 0.82). Within treatment categories, agreement was high for guideline-supported therapies (κ = 0.87) and OTC adjuncts (κ = 0.94), while alternative remedies demonstrated perfect agreement (κ = 1.00).

For psychosocial themes, agreement was strong for mental health themes (κ = 0.82) and perfect for survivor/empowerment themes (κ = 1.00) and lifestyle measures (κ = 1.00). Agreement for HS trigger identification was moderate (κ = 0.64).

Creator type and content format

Most included videos were patient-generated, accounting for approximately 90% of content, with minimal representation from HCPs. Only a small proportion of videos were created by dermatologists or other health-care providers (Table 1).

**Table 1:** Content creator type of hidradenitis suppurativa TikTok videos (n = 133). Data are presented as n (%).

Creator Type	n (%)
Patient-generated	120 (90.2)
Dermatologists	2 (1.5)
Other healthcare professionals	6 (4.5)
Advocacy groups	2 (1.5)
Other creator types	3 (2.2)
Total	133 (100)

Regarding content format, the majority of videos were testimonial in nature, while educational, promotional, advocacy-focused, and other formats comprised a much smaller proportion of content (Table 2).

**Table 2:** Video content format (n = 133). Data are presented as n (%).

Content Type	n (%)
Testimonial	106 (79.7)
Educational	9 (6.8)
Product promotion	9 (6.8)
Advocacy-focused	5 (3.8)
Other formats	4 (3.0)
Total	133 (100)

Demographics and representation

Perceived female creators comprised the majority of videos (n=120, 90%), while perceived male creators accounted for 11 videos (8%); gender could not be determined in two videos (1.5%). With respect to perceived race, most creators were coded as Black (n=75, 56%), followed by White (n=48, 36%), with smaller representation from other racial groups. Perceived race could not be determined in two videos.

Analysis of creator skin phototype demonstrated higher representation of individuals coded as Fitzpatrick IV-VI compared with Fitzpatrick I-III. A small number of videos were categorized as mixed phototype or undetermined due to insufficient visual information (Table 3). 

**Table 3:** Perceived Fitzpatrick skin type of video creators (n = 133). Data are presented as n (%).

Perceived Fitzpatrick Skin Type Category	n (%)
I-III	53 (39.8)
IV-VI	76 (57.1)
Mixed phototype	2 (1.5)
Undetermined	2 (1.5)
Total	133 (100)

Treatments

Only 32 of 133 videos (24%) referenced treatments consistent with established dermatology guidelines for HS. Among those videos, surgical or procedural interventions were most frequently discussed (n=19, 59%), followed by topical therapies (n=12, 38%), biologic agents (n=7, 22%), and systemic antibiotics (n=6, 19%). Many videos referenced more than one treatment category.

OTC adjunctive products were discussed in 38 videos (29%), while alternative or non-evidence-based remedies appeared in 25 videos (19%). Alternative remedies most commonly included non-medication herbal salves (n=15, 11%).

Psychosocial themes

Psychosocial themes were highly prevalent across HS-related TikTok content. Survivor or empowerment messaging appeared in 117 videos (88%), often emphasizing resilience, coping, self-acceptance, and encouragement. Mental health themes were present in 48 videos (36%), including references to stigma, emotional burden, emotional self-worth, anxiety, and depression.

Lifestyle-related content was present in 52 videos (39%), most commonly focusing on wound care practices and dietary considerations. Mentions of potential HS triggers occurred in 17 videos (13%), with diet and stress cited most frequently.

Engagement metrics

Engagement rates were calculated as the sum of likes, comments, saves, and shares divided by total views. Independent Welch’s t-tests showed no significant difference in engagement rates between HCP-generated and non-HCP-generated videos or between videos featuring creators coded as Fitzpatrick I-III versus Fitzpatrick IV-VI (Table 4). 

**Table 4:** Welch’s t-test comparisons of engagement rates by creator characteristics. Engagement rate was calculated as the sum of likes, comments, saves, and shares divided by total views. Comparisons were performed using independent Welch’s t-tests. Statistical significance was defined as p < 0.05.

Comparison	Group 1	Group 2	t statistic	df	p-value
Creator type	HCP-generated videos	Non-HCP-generated videos	-0.81	10.81	0.43
Creator Fitzpatrick skin type	Fitzpatrick I–III	Fitzpatrick IV–VI	-1.55	123.83	0.12

One-way ANOVA showed no significant difference in engagement rates across content formats, including educational, testimonial, and product recommendation videos (Table 5).

**Table 5:** One-way ANOVA comparing engagement rates across content formats. Engagement rate was calculated as the sum of likes, comments, saves, and shares divided by total views. Comparisons across content formats were performed using one-way analysis of variance. Statistical significance was defined as p < 0.05.

Variable compared	Groups included	F statistic	df	p-value
Engagement rate by content format	Educational, testimonial, and product recommendation videos	0.08	2, 118	0.93

## Discussion

This cross-sectional analysis of 133 TikTok videos on HS revealed several important findings. TikTok content on HS was overwhelmingly patient-generated (n=120, 90%), predominantly testimonial in nature (n=106, 80%), and largely created by women (n=120, 90%) and individuals with SOC (n=76, 57%). Psychosocial themes, such as empowerment (n=117, 88%), mental health (n=48, 36%), and lifestyle factors (n=52, 39%), were highly prevalent, whereas guideline-supported treatments were referenced in only 24% of videos (n=32). Notably, engagement metrics did not significantly differ by creator type, Fitzpatrick skin type, or content category.

The findings of this study are consistent with prior research showing that patients with HS turn to online social media platforms to seek clinical support, engage in community, and share experiences, with patient-generated content dominating online communities, such as Reddit, Facebook, and Instagram [9,19]. However, most existing studies have analyzed elements of treatment options, skin color, mental health burden, and empowerment in isolation. By concurrently examining these elements, this study provides an integrated approach to HS discourse on TikTok and strengthens the coherence within the current body of evidence.

Several important implications emerge for clinical practice and patient education. The predominance of patient-generated testimonial content emphasizes the need for dermatologists and healthcare providers treating patients with HS to engage more actively on social media platforms to offer accurate and guideline-supported information. In addition, given the strong representation of women and individuals with SOC, there is a valuable opportunity for targeted community outreach with a culturally sensitive educational approach. Moreover, the frequent discussion of alternative treatments and adjuncts suggests that clinicians should routinely inquire about social media-acquired knowledge and provide clarification, when necessary, to avoid potential harm. The strong presence of psychosocial and mental health themes highlighted the importance of integrating mental health screening into HS care to provide supportive resources using trauma-sensitive communication.

Future studies should examine longitudinal trends to understand how content and engagement patterns evolve and develop over time, especially as more HCPs have started to use short-form content for patient education. Future studies should also incorporate qualitative interviews with creators and audiences to provide deeper insight into how patients interpret and act on the information they gather online. Additionally, comparative analyses of SOC representation across multiple digital platforms may further clarify whether representation and information quality vary by social media platform.

Several limitations should be considered while interpreting these findings. TikTok’s algorithm curates top videos based on popularity; therefore, the sample may not fully capture the diversity of HS-related content on this platform. This introduces potential selection bias, as videos prioritized by the platform’s algorithm may not be representative of all HS-related TikTok content. This study analyzed high-engagement videos from community-leaning hashtags, which may overrepresent patient narratives and underrepresent less visible perspectives such as HCP content. In addition, coding of Fitzpatrick skin type relied on perceived appearance and may have been influenced by lighting or filters, although inter-rater reliability was high. Furthermore, although predefined coding definitions and inter-rater reliability testing were used, content coding may still involve subjective interpretation. Small subgroup sizes (e.g., dermatologist-created content) limited some statistical comparisons. Additionally, the short-form nature of TikTok introduces the risk of misclassification as important contextual information may not be provided. Lastly, engagement metrics on TikTok fluctuate rapidly, and our study only reflects a single time point rather than longitudinal trends. Because TikTok content and ranking algorithms change over time, the exact video sample may not be reproducible at a later date.

## Conclusions

This cross-sectional analysis of top-ranked HS-related TikTok videos under selected hashtags suggests that highly visible HS content is largely shaped by patients with HS and strongly characterized by testimonial storytelling and psychosocial themes, particularly among women and individuals with SOC. Guideline-supported treatment discussions by healthcare providers and dermatologists were comparatively limited in this sample, and engagement did not significantly differ by creator type, Fitzpatrick skin type, or selected content characteristics. These findings highlight both the value and the challenges of TikTok as a venue for HS education, underscoring opportunities for clinician participation and culturally responsive communication.
